# WT1基因突变对急性髓系白血病患者预后的影响

**DOI:** 10.3760/cma.j.cn121090-20251222-00608

**Published:** 2026-05

**Authors:** 谕 曾, 岩 惠, 琳 颜, 秋云 房, 艳 李, 颖 王, 本法 宫, 云涛 刘, 承彩 国, 寿芸 李, 冬 林, 凯奇 刘, 广吉 张, 春林 周, 晓媛 弓, 少伟 邱, 兵城 刘, 迎 王, 营昌 秘, 建祥 王, 辉 魏

**Affiliations:** 1 中国医学科学院血液病医院（中国医学科学院血液学研究所），血液与健康全国重点实验室，国家血液系统疾病临床医学研究中心，细胞生态海河实验室，天津 300020 State Key Laboratory of Experimental Hematology, National Clinical Research Center for Blood Diseases, Haihe Laboratory of Cell Ecosystem, Institute of Hematology & Blood Diseases Hospital, Chinese Academy of Medical Sciences & Peking Union Medical College, Tianjin 300020, China; 2 天津医学健康研究院，天津 301600 Tianjin Institutes of Health Science, Tianjin 301600, China

**Keywords:** 白血病，髓系，急性, 基因，WT1, 基因突变, 预后, Leukemia, myeloid, acute, Gene, WT1, Gene mutation, Prognosis

## Abstract

**目的:**

探究WT1基因突变对急性髓系白血病（AML）患者预后的影响。

**方法:**

本研究为回顾性队列研究。纳入1 583例2015年1月至2024年3月中国医学科学院血液病医院收治的AML患者，其中WT1野生型患者1 437例，WT1突变型患者146例（9.2％）。分析WT1突变型患者和野生型患者的临床特征和基因突变谱，根据2022年欧洲白血病网（ELN）风险分层和AML主要突变亚型分组对WT1突变型和野生型患者进行生存分析，观察总生存（OS）期、无复发生存（RFS）期和无事件生存（EFS）期。

**结果:**

WT1突变型和野生型患者在临床特征上差异无统计学意义（均*P*>0.05）。WT1突变型患者展示出独特的基因突变谱，常与CEBPA^bZIP^（30.1％对15.0％，*P*<0.001）和FLT3-ITD（30.1％对19.8％，*P*＝0.036）共突变，较少与NPM1（11.0％对20.4％，*P*＝0.068）和RUNX1::RUNX1T1（4.8％对19.2％，*P*<0.001）发生共突变。生存分析显示，在整体队列和各2022年ELN风险分层患者中，WT1突变型和野生型两组患者的OS、RFS和EFS差异均无统计学意义（均*P*>0.05）。WT1突变对各个AML突变亚型的预后均无明显影响，但是WT1和CEBPA^bZIP^共突变患者显示出更差的RFS（*P*＝0.042）和EFS（*P*＝0.017）。将年龄、性别、AML常见基因突变等临床因素纳入多因素Cox回归分析中，结果显示WT1不是影响预后的独立因素（OS：*P*＝0.209，*HR*＝0.796，95％*CI*：0.558～1.137；RFS：*P*＝0.390，*HR*＝1.149，95％*CI*：0.837～1.579；EFS：*P*＝0.180，*HR*＝1.192，95％*CI*：0.922～1.542）。

**结论:**

尽管WT1是AML的常见突变基因，但在本队列患者中，其存在与不良预后无显著相关性，也非独立的预后风险因素。

## Abstract (en)

**Objective:**

To investigate the prognostic impact of WT1 mutations in patients with acute myeloid leukemia（AML）.

**Methods:**

This was a retrospective cohort study. A total of 1 583 patients with AML who were admitted to the Institute of Hematology and Blood Diseases Hospital, Chinese Academy of Medical Sciences and Peking Union Medical College, from January 2015 to March 2024 were enrolled in the study, including 1 437 WT1 wild-type patients and 146（9.2％）carrying WT1 mutations. Clinical characteristics and gene mutation profiles were comprehensively compared between WT1-mutated and wild-type cohorts. Survival analyses were performed on WT1-mutant and WT1 wild-type patients in accordance with the 2022 ELN risk classification and major AML mutation subgroups. The primary outcome measures included overall survival（OS）, relapse-free survival（RFS）, and event-free survival（EFS）.

**Results:**

No statistically significant difference in clinical characteristics was found between WT1-mutant and WT1 wild-type patients（all *P*>0.05）. In addition, WT1-mutant AML displayed a unique genomic alteration profile, which is characterized by high frequencies of CEBPA^bZIP^（30.1％ *vs* 15.0％, *P*<0.001）and FLT3-ITD（30.1％ *vs* 19.8％, *P*＝0.036）, along with a reduced incidence of NPM1（11.0％ *vs* 20.4％, *P*＝0.068）and RUNX1::RUNX1T1（4.8％ *vs* 19.2％, *P*<0.001）. Survival analysis of the entire cohort and within the 2022 ELN risk classification revealed no significant differences in OS, RFS, or EFS between WT1-mutated and wild-type groups（all *P*>0.05）. Although WT1 mutations had no significant prognostic impact on different AML subgroups, WT1 mutations were associated with inferior RFS（*P*＝0.042）and EFS（*P*＝0.017）in patients co-mutated with CEBPA^bZIP^. Clinical factors, including age, sex, and common AML mutation genes, were incorporated into multivariate Cox regression analyses, which consistently showed that WT1 was not an independent prognostic risk factor（OS: *P*＝0.209, *HR*＝0.796, 95％*CI*: 0.558–1.137; RFS: *P*＝0.390, *HR*＝1.149, 95％*CI*: 0.837–1.579; EFS: *P*＝0.180, *HR*＝1.192, 95％*CI*: 0.922–1.542）.

**Conclusion:**

Although WT1 is a common mutation gene in AML, it was not associated with poor prognosis and was not an independent prognostic risk factor in our cohort.

肾母细胞瘤基因1（WT1）是一种编码锌指转录因子的基因，其在急性髓系白血病（AML）患者中突变频率为10％～15％[1]–[3]。该基因的突变形式（如核苷酸插入或无义突变）常导致其编码蛋白功能丧失，从而削弱其肿瘤抑制功能，促进白血病的发生和发展[2]。既往研究显示，WT1突变与较高的可检测残留病（MRD）阳性率、较高的复发风险和较低的总生存（OS）率相关[4]–[8]。早期研究表明，WT1表达异常的AML患者（阴性或高表达）复发率更高，在未接受造血干细胞移植干预的情况下OS更差；而移植后，WT1阴性和中等表达及高表达患者的生存结局无显著差异[9]。另有研究证实，化疗期间WT1表达水平的下调，与患者生存预后的改善密切相关[10]。

先前对WT1突变在AML中不良预后作用的探讨，多集中于伴NPM1、FLT3-ITD、CEBPA等常见突变患者，WT1在其他AML亚型中的预后意义尚未得到充分阐明。为系统评价WT1突变在AML中的临床价值，本研究纳入1 583例AML患者展开队列研究，比较WT1突变型和野生型患者的临床特征，并结合2022年欧洲白血病网（ELN）风险分层和AML分子亚型进行生存分析。本研究假设WT1突变的预后影响并非在所有AML亚型中均一，可能因不同共突变基因及ELN风险分层而异。通过上述分析，本研究旨在系统评估WT1突变对AML预后的影响，从而为预后分层提供更为可靠的依据。

## 病例与方法

1. 研究对象：本研究为回顾性队列研究。选取分析2015年1月至2024年3月在中国医学科学院血液病医院连续治疗的非急性早幼粒细胞白血病（non-APL）AML患者。所有患者按照WHO2016髓系肿瘤分类标准进行诊断。本队列纳入标准具体如下：①年龄14～65岁；②接受强化诱导化疗；③接受中、高剂量阿糖胞苷巩固治疗；④具有完整的基因突变谱数据。本研究遵循《赫尔辛基宣言》原则并获得中国医学科学院血液病医院伦理委员会批准（批件号：NSFC2023015-EC-2），所有参与者均签署了关于治疗方案和生物样本使用的知情同意书。

2. 治疗：所有患者均接受了强化诱导治疗，治疗方案为标准剂量（100 mg·m^−2^·d^−1^，第1～7天，12 h静脉输注）或中剂量阿糖胞苷［100 mg·m^−2^·d^−1^，第1～4天，12 h静脉输注；1 g·m^−2^·（12 h）^−1^，第5～7天，3 h静脉输注］联合蒽环类药物，联用或不联用高三尖杉酯碱（HHT）。患者获得完全缓解（CR）后接受3个周期中、高剂量阿糖胞苷巩固治疗。对于具有中高危遗传学风险或存在持续MRD，且具备合适供者及可耐受移植的患者，推荐行异基因造血干细胞移植（allo-HSCT）。

3. 二代测序（NGS）：采用骨髓单个核细胞来源的基因组DNA，利用聚合酶链反应（PCR）对其进行扩增，并通过二代测序技术开展突变分析。测序在Illumina NovaSeq 6000平台上进行，所有检测基因均与血液系统恶性肿瘤高度相关，靶向panel涵盖WT1等关键基因。文库构建采用核糖体RNA去除法，测序数据的平均覆盖度达98％，平均测序深度为1 000×～2 000×。所有测序结果均基于GRCh37/hg19参考基因组进行比对，并利用COSMIC、ClinVar、HGMD、ExAC、ESP6500、GnomAD及dbSNP等多个数据库对单核苷酸变异（SNVs）及插入/缺失（Indels）进行注释和筛选。使用R 4.4.2的complexheatmap R包对突变谱进行可视化分析。

4. 研究终点定义：OS期指从入院起至任何原因导致死亡的时间，失访患者在末次随访时删失。无事件生存（EFS）期指从入院起至治疗失败、疾病复发或任何原因死亡中先发生者为止的时间间隔。无复发生存（RFS）期指从首次获得CR之日起至复发、死亡或末次随访的时间。

5. 随访：随访方式为电话随访，随访截止时间为2025年3月6日。

6. 统计学处理：分类变量以频数和百分比描述，组间比较视数据情况采用卡方检验或Fisher精确检验。连续变量以*M*（*IQR*）表示，组间差异通过Wilcoxon秩和检验进行比较。若进行多重比较，则对*P*值进行Bonferroni校正。采用Kaplan-Meier法进行OS、RFS和EFS曲线绘制，并通过Log-rank检验评估组间生存差异。通过Cox回归模型对研究队列进行多因素分析。*P*<0.05为差异具有统计学意义。本研究统计分析和绘图均使用R 4.4.2完成。

## 结果

1. 临床特征：最终有1 583例患者符合纳入标准进入研究队列。筛选流程如图1所示。队列包括146例WT1突变型患者和1 437例野生型患者。WT1突变型患者和野生型患者在性别、年龄、WBC、HGB、PLT上差异均无统计学意义（均*P*>0.05）。根据2022年ELN风险分层，WT1突变型患者在低危、中危和高危组中的分布为43.8％（64/146）、39.7％（58/146）和16.4％（24/146）；WT1野生型患者的分布为50.1％（720/1 437）、27.8％（399/1 437）和22.1％（318/1 437）。经过2个周期诱导治疗后，WT1突变型患者和野生型患者的CR率分别为80.8％（118/146）和84.1％（1 209/1 437）。在达到首次CR的患者中，WT1突变型患者与野生型患者接受allo-HSCT的比例接近，分别为26.7％（39/146）和26.4％（379/1 437）。此外，WT1突变型和野生型患者的形态学复发率分别为30.1％（44/146）和27.3％（393/1 437）（表1）。

**图1:**
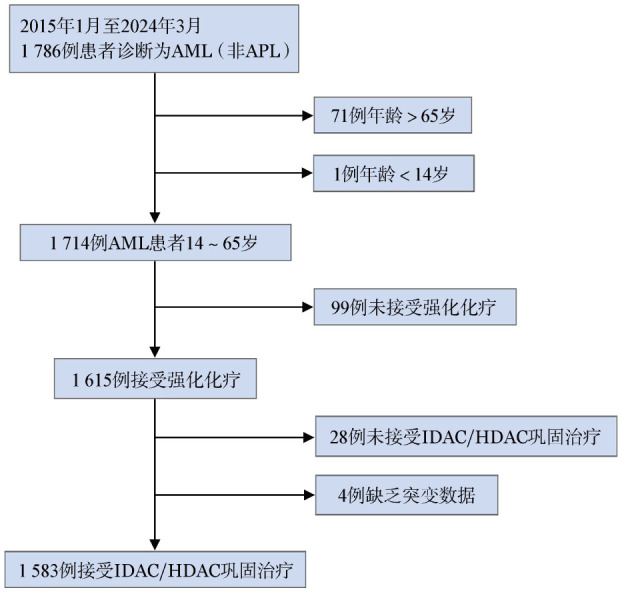
纳入队列的AML患者筛选流程图 **注** AML：急性髓系白血病；APL：急性早幼粒细胞白血病；IDAC/HDAC：中剂量/高剂量阿糖胞苷

**表1:** 研究队列的临床特征

特征	总体	WT1突变型（146例）	WT1野生型（1 437例）	*P*值
性别［例（％）］				0.207
男	841（53.1）	64（43.8）	777（54.1）	
女	742（46.9）	82（56.2）	660（45.9）	
年龄［岁，*M*（*IQR*）］	40.1（30.0, 51.0）	39.2（31.0, 48.8）	40.2（30.0, 51.0）	1.000
年龄［例（％）］				
<20 岁	135（8.5）	12（8.2）	123（8.6）	
20～≤40 岁	611（38.6）	65（44.5）	546（38.0）	
40～≤60 岁	746（47.1）	63（43.2）	683（47.5）	
>60 岁	91（5.7）	6（4.1）	85（5.9）	
WBC［×10⁹/L，*M*（*IQR*）］	33.8（4.2, 43.6）	33.5（5.4, 43.6）	33.8（4.0, 43.6）	1.000
HGB［g/L，*M*（*IQR*）］	85.3（70.0, 99.0）	86.6（70.6, 101.3）	85.2（70.0, 98.0）	1.000
PLT［×10⁹/L，*M*（*IQR*）］	61.6（25.0, 73.0）	59.2（22.0, 71.0）	61.8（25.0, 73.0）	1.000
2022年ELN风险分层［例（％）］				0.074
低危	784（49.5）	64（43.8）	720（50.1）	
中危	457（28.9）	58（39.7）	399（27.8）	
高危	342（21.6）	24（16.4）	318（22.1）	
诱导化疗2个周期内获得完全缓解［例（％）］	1 327（83.8）	118（80.8）	1 209（84.1）	1.000
形态学复发［例（％）］	437（27.6）	44（30.1）	393（27.3）	1.000
首次完全缓解行造血干细胞移植［例（％）］	418（26.4）	39（26.7）	379（26.4）	1.000

2. 基因突变谱：对WT1突变型和野生型患者的基因突变谱进行比较分析发现，两组在基因突变的发生频率上差异有统计学意义。在WT1突变型患者中，常见的基因突变有CEBPA^bZIP^（44例，30.1％）、FLT3-ITD（44例，30.1％）、NRAS（42例，28.8％）、NPM1（16例，11.0％）、TET2（15例，10.3％）和CBFB::MYH11（13例，8.9％）（图2）。而在WT1野生型患者中，常见的基因突变有NRAS（353例，24.6％）、NPM1（293例，20.4％）、FLT3-ITD（284例，19.8％）、RUNX1::RUNX1T1（276例，19.2％）和CEBPA^bZIP^（215例，15.0％）（**扫描本文首页二维码查阅附图1**）。

**图2:**
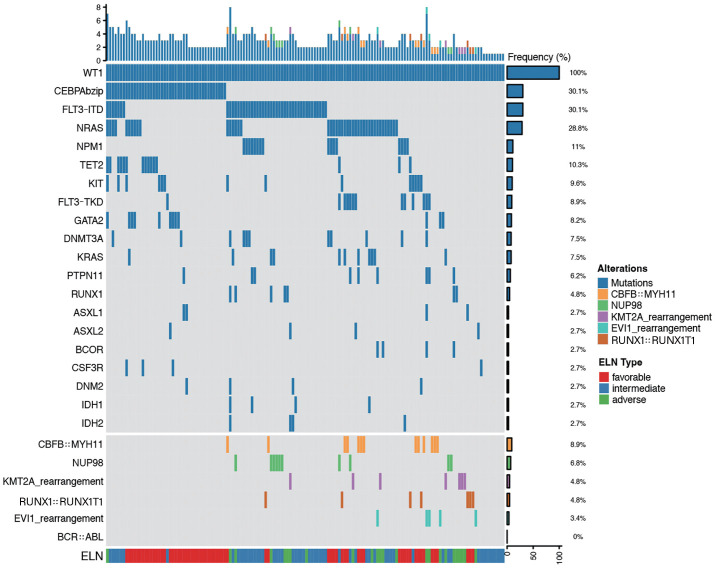
WT1突变型急性髓系白血病患者的基因突变频谱图（每列蓝格代表1例患者）

组间比较显示，WT1突变型患者CEBPA^bZIP^（*P*<0.001）和FLT3-ITD（*P*＝0.036）的共突变频率显著高于野生型患者，而NPM1（*P*＝0.068）的共突变频率则呈现偏低的趋势。在融合基因方面，WT1突变型患者的NUP98重排（*P*<0.001）和EVI1重排（*P*＝0.050）的共突变频率更高，而RUNX1::RUNX1T1（*P*<0.001）的共突变频率则明显低于野生型患者。两组间在CBFB::MYH11和KMT2A重排的发生率上差异无统计学意义（表2）。

**表2:** WT1突变型和WT1野生型急性髓系白血病患者基因突变频率［例（％）］

共突变基因	总体	WT1突变型	WT1野生型	*P*值
CEBPA^bZIP^	259（16.4）	44（30.1）	215（15.0）	<0.001
FLT3-ITD	328（20.7）	44（30.1）	284（19.8）	0.036
NPM1	309（19.5）	16（11.0）	293（20.4）	0.068
RUNX1::RUNX1T1	283（17.9）	7（4.8）	276（19.2）	<0.001
CBFB::MYH11	124（7.8）	13（8.9）	111（7.7）	1.000
NUP98重排	26（1.6）	10（6.8）	16（1.1）	<0.001
KMT2A重排	123（7.8）	7（4.8）	116（8.1）	1.000
EVI1重排	14（0.9）	5（3.4）	9（0.6）	0.050

3. WT1突变对预后的影响：我们进一步评估了WT1突变对AML患者预后的影响。在整个队列中，WT1突变型和野生型患者的OS（*P*＝0.170）、RFS（*P*＝0.340）和EFS（*P*＝0.110）差异均无统计学意义（图3A～C）。WT1突变型和野生型患者的5年OS率分别为（66.9±5.9）％和（59.9±1.5）％（*HR*＝0.78, 95％*CI*：0.55～1.11, *P*＝0.169）；5年RFS率分别为（49.7±8.1）％和（55.3±1.7）％（*HR*＝1.16, 95％*CI*: 0.85～1.59, *P*＝0.337）；5年EFS率分别为（41.2±6.9）％和（48.4±1.6）％（*HR*＝1.23, 95％*CI*: 0.95～1.58, *P*＝0.109）。

**图3:**
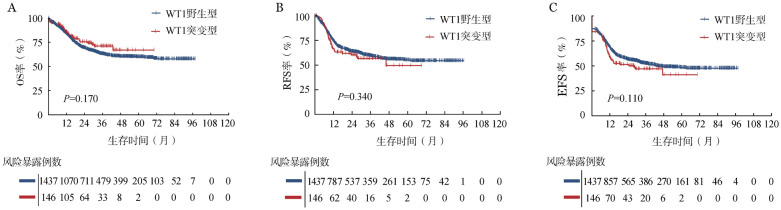
WT1突变型和野生型组急性髓系白血病（AML）患者的总生存（OS）（A）、无复发生存（RFS）（B）、无事件生存（EFS）（C）曲线

按风险分层进行生存分析显示，WT1突变在低危组（OS: *P*＝0.110, RFS: *P*＝0.084, EFS: *P*＝0.110）（图4A～C）、中危组（OS: *P*＝0.900, RFS: *P*＝0.770, EFS: *P*＝0.790）（图4D～F）和高危组（OS: *P*＝0.084，RFS: *P*＝0.580，EFS: *P*＝0.760）（图4G～I）中均未对预后产生显著影响。

**图4:**
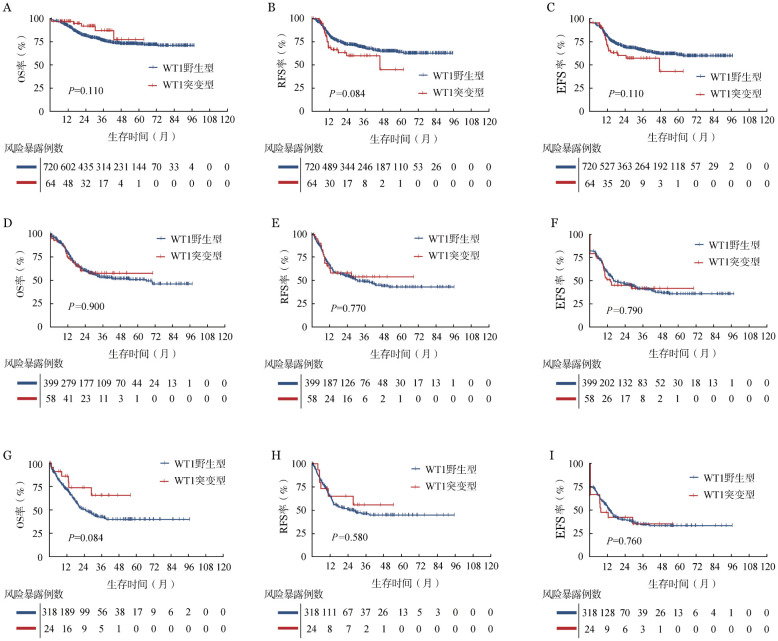
各2022年欧洲白血病网（ELN）风险分层中WT1野生型和突变型急性髓系白血病（AML）患者的生存曲线 **A～C** 在低危患者中两组的总生存（OS）、无复发生存（RFS）、无事件生存（EFS）曲线；**D～F** 在中危患者中两组的OS、RFS、EFS曲线；**G～I** 在高危患者中两组的OS、RFS、EFS曲线

进一步针对不同AML亚型的分析表明，与CEBPA^bZIPmut^WT1^wt^的患者相比，CEBPA^bZIPmut^WT1^mut^患者的RFS（*P*＝0.042）和EFS（*P*＝0.017）更差，两者OS差异无统计意义（*P*＝0.670）（图5A～C）。CEBPA^bZIPmut^WT1^mut^和CEBPA^bZIPmut^WT1^wt^患者的5年RFS率分别为（22.5±16.5）％和（57.6±4.0）％（*HR*＝1.67, 95％*CI*: 1.01～2.73, *P*＝0.044）；5年EFS率分别为（21.3±15.6）％和（56.0±3.9）％（*HR*＝1.75, 95％*CI:* 1.10～2.79, *P*＝0.018）。WT1突变未对RUNX1::RUNX1T1（OS: *P*＝0.190, RFS: *P*＝0.960, EFS: *P*＝0.880）、CBFB::MYH11（OS: *P*＝0.430, RFS: *P*＝0.320, EFS: *P*＝0.700）、NPM1突变（OS: *P*＝0.810, RFS: *P*＝0.960, EFS: *P*＝0.860）和KMT2A重排（OS: *P*＝0.290, RFS: *P*＝0.650, EFS: *P*＝0.990）的AML患者的生存预后产生不良影响（**扫描本文首页二维码查阅附图2～5**）。同样，在伴有骨髓增生异常相关改变的AML（AML-MRC）患者中，WT1突变与OS、RFS和EFS均无显著相关性（OS: *P*＝0.420, RFS: *P*＝0.330, EFS: *P*＝0.170）（**扫描本文首页二维码查阅附图6**）。此外，在FLT3-ITD突变患者中，WT1共突变患者与野生型患者相比，OS（*P*＝0.870）、RFS（*P*＝0.720）和EFS（*P*＝0.530）差异均无统计学意义（**扫描本文首页二维码查阅附图7**）。

**图5:**
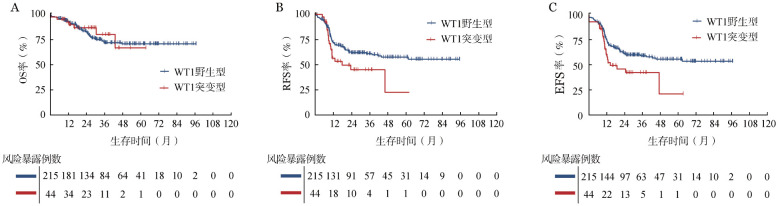
CEBPA^bZIP^突变患者中WT1突变型和野生型组的总生存（OS）（A）、无复发生存（RFS）（B）、无事件生存（EFS）（C）曲线

为了明确影响预后的独立因素，我们将年龄、性别、其他临床特征和突变类型纳入多因素Cox回归分析，结果显示WT1突变不是独立预后因素（表3）。

**表3:** 急性髓系白血病（AML）预后的多因素Cox回归分析

变量	总生存期		无复发生存期		无事件生存期	
	*HR*（95％*CI*）	*P*值	*HR*（95％*CI*）	*P*值	*HR*（95％*CI*）	*P*值
年龄	1.024（1.016~1.031）	<0.001	1.010（1.002~1.017）	0.009	1.008（1.002~1.014）	0.007
WBC	1.003（1.002~1.005）	<0.001	1.003（1.002~1.005）	<0.001	1.004（1.003~1.005）	<0.001
性别	1.157（0.969~1.382）	0.108	1.090（0.910~1.305）	0.349	1.177（1.012~1.370）	0.034
CR_1_ HSCT	0.333（0.261~0.423）	<0.001	0.278（0.218~0.354）	<0.001	0.259（0.210~0.319）	<0.001
WT1突变	0.796（0.558~1.137）	0.209	1.149（0.837~1.579）	0.390	1.192（0.922~1.542）	0.180
NPM1突变	0.518（0.403~0.667）	<0.001	0.516（0.394~0.677）	<0.001	0.435（0.348~0.543）	<0.001
FLT3-ITD突变	1.565（1.261~1.942）	<0.001	1.642（1.308~2.063）	<0.001	1.636（1.360~1.968）	<0.001
CEBPA^bZIP^突变	0.337（0.248~0.459）	<0.001	0.550（0.419~0.722）	<0.001	0.373（0.294~0.473）	<0.001
RUNX1::RUNX1T1	0.473（0.355~0.629）	<0.001	0.505（0.381~0.668）	<0.001	0.379（0.298~0.482）	<0.001
CBFB::MYH11	0.245（0.152~0.397）	<0.001	0.177（0.107~0.292）	<0.001	0.158（0.103~0.243）	<0.001
KMT2A重排	1.782（1.322~2.401）	<0.001	1.867（1.352~2.578）	<0.001	1.289（0.982~1.691）	0.067
AML-MRC	1.132（0.882~1.454）	0.329	0.959（0.721~1.276）	0.774	1.177（0.954~1.452）	0.129

**注** CR_1_ HSCT：首次完全缓解期造血干细胞移植；AML-MRC：伴有骨髓增生异常相关改变的AML

## 讨论

本临床队列研究旨在明确WT1突变在AML患者中的预后价值。既往多项研究表明WT1突变与白血病发生、疾病进展和临床预后密切相关[11]–[12]，且被认为是影响预后的独立因素[5],[8],[13]。然而本研究在评估WT1突变的预后意义时，却得到了不同结论：WT1突变并非影响AML患者临床预后的独立因素。具体而言，无论是在整体队列中，还是在不同ELN风险分层中或各个AML突变亚型中，WT1突变型和野生型患者的OS、RFS和EFS均未观察到统计学差异，仅在CEBPA^bZIP^和WT1共突变的AML患者中观察到较差的RFS和EFS；此外，两组患者的CR率和形态学复发率亦无显著不同。

为阐释本研究结论与既往报道存在差异的潜在原因，我们系统对比了本队列与既往研究队列的基线特征与治疗方案。在年龄分布上，本队列中WT1突变型和野生型患者的中位年龄差异无统计学意义，这一特征与Yu等[14]的研究结果一致；而Hou等[13]的研究队列中WT1突变型患者的中位年龄也与本队列相近。治疗方案层面，各研究队列均采用以阿糖胞苷联合蒽环类药物为基础的诱导化疗方案，排除了治疗策略差异对预后评估的干扰。值得注意的是，与既往研究相比，本研究队列样本量更大，且具备完整、长期的随访资料，这为在更充足的样本量和更长的时间维度下精准评估WT1突变的预后价值提供了优势。进一步分析表明，不同研究队列中的WT1突变的共突变背景异质性可能是导致其预后意义结论不一致的原因。例如，Hou等[13]–[14]的研究显示，WT1与NPM1或CEBPA^bZIP^共突变时，患者可能呈现出更好的生存结局[13]；而Yu等[14]的研究则进一步将WT1突变型患者按照表达水平分为高表达和低表达组，发现在WT1低表达组中NPM1或FLT3-ITD突变预示着更差预后，而在高表达组中，NPM1突变则与更好的预后相关。这提示我们WT1突变的预后影响可能受到共突变状态和自身表达水平的调控。

本研究对WT1突变AML患者的基因突变谱进行了进一步揭示，WT1突变患者更常伴随CEBPA^bZIP^、FLT3-ITD和NRAS基因共突变，其中CEBPA^bZIP^和FLT3-ITD的突变频率显著较高，而与NPM1和RUNX1::RUNX1T1的共突变频率较低，这些结论与先前文献报道的结果相符合[15]–[17]。我们观察到在CEBPA^bZIP^突变患者中，WT1共突变患者的RFS和EFS均差于WT1野生型患者，与Tien等[18]–[20]的报道一致。研究表明，CEBPA^bZIP^和WT1共突变可能通过协同作用，导致干扰素信号通路（如IFN-α/γ反应）异常激活和氧化磷酸化代谢重编程（如线粒体复合物I/V相关基因表达上调），从而引起免疫和代谢功能失调，进而增强白血病干细胞的存活能力与化疗耐药性，最终导致患者OS期显著缩短[21]。有研究显示，在AML PDX模型中敲除WT1，能够在一定程度上增加阿糖胞苷的抗肿瘤效果；并且，在竞争性极限稀释实验中，也发现WT1敲除后能减少白血病起始细胞的数量[22]。关于WT1突变AML患者中NPM1共突变的频率和预后意义仍存在争议。Irena等[23]的研究显示AML患者中WT1和NPM1的共突变频率较高，而在本研究和Heiko等[24]的研究中却发现WT1突变患者的NPM1突变频率反而降低。Wang等[15]提出，NPM1突变可能通过CD34相关调控通路来上调WT1的表达，Xu等[10]也报道，NPM1突变和WT1高表达之间存在显著相关性，提示此类共突变可能对诱导化疗的应答产生不利影响。尽管如此，本研究结果显示在NPM1突变的背景下WT1是否突变并未对OS、RFS和EFS产生影响。近期研究显示WT1不仅是一个预后相关的生物标志物，更逐渐成为AML免疫治疗领域一个有前景的免疫靶点。例如，WT1抗原在成人和儿童AML患者中表达高度重叠，可作为跨越年龄界限的共性免疫治疗靶点，为开发更高效、低毒性的免疫治疗提供新方向[25]。

本研究存在一定的局限性。第一，本项单中心回顾性研究可能存在固有的选择和地区偏倚，研究结论仍需在多中心、前瞻性队列中进一步验证；第二，本研究未纳入MRD动态监测数据，MRD是AML重要的独立预后指标，而WT1作为MRD的潜在检测标志物，该数据的缺失可能限制了对WT1突变预后影响的全面分析；第三，部分AML亚型的样本量相对有限，未来仍需扩大各亚型的样本规模，以更精确地评估WT1突变在不同遗传背景下的预后意义。

综上所述，在本研究队列中，WT1虽然是AML常见突变基因，但是WT1突变与不良预后无显著相关性，并且也不是独立的预后因素。

## Supplementary Material


