# Supplementary figures and images for: Prognostic impact of WT1 mutations in patients with acute myeloid leukemia
Source: Zhonghua Xue Ye Xue Za Zhi. 2026 May;47(5):442–9. [Article in Chinese] doi: 10.3760/cma.j.cn121090-20251222-00608 (PMC13416534; doi:10.3760/cma.j.cn121090-20251222-00608)

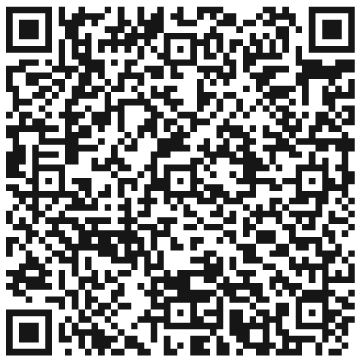

Supplement: Supplementary file 1 [file cjh-47-05-442-g006.tif]
